# Hydroxychloroquine induced QT prolongation in a schizoaffective patient being treated for a COVID-19 infection: A Case Report

**DOI:** 10.1192/j.eurpsy.2022.1840

**Published:** 2022-09-01

**Authors:** R. Amazan, P. Korenis, S. Gunturu

**Affiliations:** 1 Bronxcare health system, Psychiatry, Bronx, United States of America; 2 Bronx Care Health System-Affiliated with the Icahn School of Medicine at Mount Sinai, Psychiatry, New York, United States of America

**Keywords:** Hydroxychloroquine, schizoaffective, QT prolongation, Covid-19

## Abstract

**Introduction:**

Hydroxychloroquine an antimalarial medication has been approved in March 2020 by FDA for treatment of hospitalized patient with COVID-19 infection. Even thus, its efficacy has been controversial, it still being used worldwide. This medication also causes some serious side effects. Here we present a case of a woman with a very long history of treatment resistant schizoaffective disorder, on clozapine, who develops QT prolongation after receiving hydroxychloroquine for the treatment of COVID-19 infection.

**Objectives:**

Despite the controversy, this case aims to shed light on the importance of monitoring QTc via EKG in patient receiving hydroxychloroquine^7^. More importantly to avoid antipsychotic while patient is receiving this medication since both hydroxychloroquine and most antipsychotic can increase QTc.

**Methods:**

This case report was written by reviewing chart of the patient and also via direct interaction and interviews with the patient.

**Results:**

This case report showed and increased in QTc interval after receiving hydroxychloroquine, which is also reported by others including Moussa Sleh et al in their article on Effect of Chloroquine, Hydroxychloroquine, and Azithromycin on the Corrected QT Interval in Patients With SARS-CoV-2 Infection^4^. The increase in Qtc could have been worse if Clozapine was not stopped during this time.

**Conclusions:**

COVID-19 pandemic has caused more than 700000 deaths around the globe and more than 150000 deaths in the United States of America. Psychiatric patients are also getting hospitalized and receiving treatment with hydroxychloroquine. Holding anti-psychotics and monitoring of QTc via EKG resulted crucial in limiting the adverse effect of QT prolongation of both medications.

**Disclosure:**

No significant relationships.

